# Optical Biopsy of the Upper GI Tract Using Fluorescence Lifetime and Spectra

**DOI:** 10.3389/fphys.2020.00339

**Published:** 2020-05-13

**Authors:** Zhaojun Nie, Shu-Chi Allison Yeh, Michelle LePalud, Fares Badr, Frances Tse, David Armstrong, Louis W. C. Liu, M. Jamal Deen, Qiyin Fang

**Affiliations:** ^1^School of Biomedical Engineering, Faculty of Engineering, McMaster University, Hamilton, ON, Canada; ^2^Advanced Microscopy Program, Center for Systems Biology and Wellman Center for Photomedicine, Massachusetts General Hospital, Harvard Medical School, Boston, MA, United States; ^3^Division of Gastroenterology and Farncombe Family Digestive Health Research Institute, Department of Medicine, McMaster University, Hamilton, ON, Canada; ^4^Division of Gastrointestinal Diseases, Department of Medicine, University of Toronto, Toronto, ON, Canada; ^5^Department of Electrical and Computer Engineering, Faculty of Engineering, McMaster University, Hamilton, ON, Canada; ^6^Department of Engineering Physics, Faculty of Engineering, McMaster University, Hamilton, ON, Canada

**Keywords:** diffuse reflectance, esophageal cancer, fiber optic probe, fluorescence spectroscopy, gastrointestinal, optical biopsy, time-resolved fluorescence

## Abstract

Screening and surveillance for gastrointestinal (GI) cancers by endoscope guided biopsy is invasive, time consuming, and has the potential for sampling error. Tissue endogenous fluorescence spectra contain biochemical and physiological information, which may enable real-time, objective diagnosis. We first briefly reviewed optical biopsy modalities for GI cancer diagnosis with a focus on fluorescence-based techniques. In an *ex vivo* pilot clinical study, we measured fluorescence spectra and lifetime on fresh biopsy specimens obtained during routine upper GI screening procedures. Our results demonstrated the feasibility of rapid acquisition of time-resolved fluorescence (TRF) spectra from fresh GI mucosal specimens. We also identified spectroscopic signatures that can differentiate between normal mucosal samples obtained from the esophagus, stomach, and duodenum.

## Introduction

Malignancies in the upper gastrointestinal (GI) tract have remained prevalent in the past few decades (American Cancer Society, 2019). As of 2019, the 5-year survival rates of esophageal and stomach cancer combining all stages are 19 and 31%, respectively (American Cancer Society, 2019). Of note, the average survival rate is approximately 10-fold higher when the cancer is detected *in situ* compared to late stage diagnosis (American Cancer Society, 2019). The low survival rate is attributed to the lack of early diagnosis and effective treatment options for late stage diseases (Wilson, 2007). Taking esophageal adenocarcinoma (EAC) as an example, a typical progression path starts with chronic gastroesophageal reflux disease (GERD), which is a common condition with a prevalence of 18–28% in North America (El-Serag et al., 2014). Gastroesophageal reflux causes chronic injury to the normal stratified squamous epithelium which, in about 20% of individuals (Schlottmann et al., 2017), is replaced by columnar-lined epithelium (CLE) in the distal esophagus, a process called columnar metaplasia. If intestinal metaplasia (IM) containing goblet cells is also found, there is significant risk of further development of dysplasia and carcinoma (Wang and Sampliner, 2008). In the United States, CLE with IM is the diagnostic criterion for Barrett’s esophagus (BE), which is considered a main risk factor for EAC. It should be noted that, in the United Kingdom, BE is defined as visual evidence of CLE without IM (Shaheen et al., 2016).

The malignancy transformation process of metaplasia-dysplasia-carcinoma suggests that early detection and treatment of precancerous dysplasia holds promise to reduce the risk of cancer (Wang and Sampliner, 2008). Although gastroesophageal reflux is often symptomatic and CLE is usually visible under white light endoscopy surveillance, IM and dysplasia are generally present as focal lesions that are invisible during video endoscopy and need to be diagnosed by random 4-quadrant biopsies, taken every 1-2 cm along the length of the section of CLE, supplemented by targeted biopsies if there are any localised areas of mucosal irregularity (Wang and Sampliner, 2008). Histopathological analysis following tissue biopsy is currently the clinical gold standard for confirming the diagnosis of BE. Pathological standards for diagnosis of metaplasia and dysplasia include cytological and architectural abnormalities (Goldblum, 2003; Fiocca et al., 2011). For example, BE may show columnar metaplasia, characterized by cardiac-type or oxyntic-type epithelium with or without features of IM. The IM can, then, be further characterized as “complete” (features of small intestine mucosa: goblet cells, absorptive cells, and Paneth cells) or “incomplete” (features of both intestine and stomach mucosa—usually lacking absorptive cells and Paneth cells). Incomplete IM is considered more likely than complete IM to progress through the dysplasia-carcinoma sequence (Voltaggio et al., 2011).

Although the rationale of tissue biopsy, histopathology, and treatment followed by regular endoscopic surveillance seems reasonable and practical for early management of BE, the incidence of upper GI cancer has remained stable in the past decades (Shaheen et al., 2016). In other words, current surveillance strategies are imperfect due to several possible reasons. First, although biopsies should be taken from any areas of mucosal irregularity to improve the detection of neoplasia or malignancy, early dysplastic lesions may not be visible with white light endoscopy because these lesions can be focal and distributed irregularly, thus missed by routine random biopsies (Mastracci et al., 2009; Veitch et al., 2015). For example, it has been reported that the sensitivity of eight biopsies in a metaplasia segment away from the esophageal-gastric junction is just 68% (Fiocca et al., 2011). Second, a meta-analysis conducted in 2014 showed that, even in the presence of confirmed neoplasia, there is an 11.3% miss rate of upper GI cancers in endoscopic surveillance (Menon and Trudgill, 2014). Third, accurate diagnosis is hampered by the qualitative or semi-quantitative nature of the diagnostic criteria for neoplasia and by marked intra- and inter-observer variation in the identification of these features (Mastracci et al., 2009; Muldoon et al., 2010). Consequently, improvements are needed toward a robust detection approach for survey and classification of upper GI lesions.

Besides white light endoscopy, several specialized wide-field endoscopic approaches have been developed to assist practitioners in identifying pathological lesions, and then proceed to either endoscopic treatment or a standard tissue biopsy for confirmation. These techniques can be based on either endogenous [autofluorescence and narrow band imaging (NBI)] (Kara and Bergman, 2006) or exogenous contrast (e.g., chromoendoscopy) (Amano et al., 2005; Wong Kee Song et al., 2007). Specifically, for endogenous fluorescence, neoplastic regions are found to have degraded collagen structure, mucosal thickening that enhances blue light attenuation, angiogenesis that increases hemoglobin absorption (Wilson, 2007), and increased cell metabolism that affects a variety of coenzymes such as reduced nicotinamide adenine dinucleotide (NADH) and flavin-adenine dinucleotide (FAD) (Skala et al., 2007b). These characteristics cause an overall brownish contour and darker vascular pattern of the lesion, which is reflected in their reflectance spectra and images (Qiu et al., 2010). Many of these wide field endoscopic approaches have been adopted, to a greater or lesser extent, by some academic centers that specialize in the management of BE, but they have not been widely adopted in clinical practice. Work regarding wide field endoscopic imaging has been reviewed extensively by Wilson (2007), Lee et al. (2012), and Veitch et al. (2015).

Given the limited space inside an endoscope, these specialized wide-field imaging techniques such as fluorescence and NBI have limited spectral resolution (e.g., filter based), spectral selectivity (e.g., fixed number of spectral bands), and sensitivity/dynamic range. Point-detection techniques, in contrast to macroscopic observation, still hold advantages especially when being used in combination with wide-field modalities to explore a focal area for biopsy.

## Optical Biopsy for Upper GI Tract

Optical spectroscopy or microscopic imaging is capable of rendering spatial resolution and/or contrast beyond perception of human eyes. The term, *Optical Biopsy*, refers to point-detection techniques that enable real-time, minimally invasive diagnosis of a focal area. It can be used to guide needle biopsy and holds the promise to provide diagnosis without the need of additional histopathological examination. Advances in optical biopsy will not only mitigate sampling error issues, but also make real-time endoscopic treatment feasible, hence further reducing the mis-treatment rate of focal lesions. A list of optical biopsy technologies for GI cancer diagnosis is summarized in Table 1. It should be noted that the references cited here are representative and by no means comprehensive.

**TABLE 1:** Optical biopsy modalities for gi cancer diagnosis: listed by organ.

	**Oral/throat**	**Esophagus**	**Stomach**	**Colon**	**Pancreas**
Fluorescence spectroscopy	Rahman et al., 2010; Pierce et al., 2012; van den Berg et al., 2012; Laronde et al., 2014; Yuvaraj et al., 2014	Boerwinkel et al., 2015; Jiang et al., 2017	Murayama et al., 2012; Koizumi et al., 2013	Iftimia et al., 2012	
Confocal	Muldoon et al., 2012; Thong et al., 2012; El Hallani et al., 2013; Jabbour et al., 2013	Kiesslich et al., 2006; Kara et al., 2007; Becker et al., 2008; Dunbar et al., 2009; Dunbar and Canto, 2010; Wallace et al., 2010; Bertani et al., 2013; Canto et al., 2014	Jeon et al., 2011	Buchner et al., 2010	
Multiphoton	Pal et al., 2017	Wong et al., 2014			
Time-resolved fluorescence	Xie et al., 2012; Sun et al., 2012; Yuvaraj et al., 2014; Kanniyappan et al., 2016	Glanzmann et al., 1999; Pfefer et al., 2003		Mycek et al., 1998; Coda et al., 2014	
Imaging (FLIM)	Skala et al., 2007a; Park et al., 2010; Fatakdawala et al., 2013; Jabbour et al., 2013; Sun et al., 2013; Cheng et al., 2014; Malik et al., 2016; Pande et al., 2016	Fuhrmann et al., 2011	McGinty et al., 2010	McGinty et al., 2010	McGinty et al., 2010
Reflectance	De Veld et al., 2005; Schwarz et al., 2008; Jabbour et al., 2013; van Leeuwen-van Zaane et al., 2013; Yu et al., 2014; Einstein et al., 2016; Bailey et al., 2017	Qiu et al., 2010; Douplik et al., 2014; Lariviere et al., 2018		Baltussen et al., 2017	Zhang et al., 2017
Raman	Bergholt et al., 2012; Singh et al., 2012, 2013; Guze et al., 2015	Feng et al., 2013; Almond et al., 2014; Bergholt et al., 2014	Teh et al., 2010; Duraipandian et al., 2012		
OCT	Armstrong et al., 2006; Yang et al., 2008; Kim et al., 2009; Huo et al., 2010; Jerjes et al., 2010; Just et al., 2010; Hamdoon et al., 2013, 2016; Boadi et al., 2015; Chen et al., 2018	Bouma et al., 2000; Pitris et al., 2000; Evans et al., 2007; Testoni and Mangiavillano, 2008; Cobb et al., 2010; Suter et al., 2014; Wolfsen et al., 2015	Bouma et al., 2000; Evans et al., 2007	Terry et al., 2011; Iftimia et al., 2012	Testoni and Mangiavillano, 2008
Photoacoustic	Fatakdawala et al., 2013	Lim et al., 2015; Yang et al., 2015			

### Fluorescence-Based Techniques

When a biological molecule absorbs incident light, sometimes it is excited from a resting ground state to more energetic and unstable excited states. Fluorescence emission then occurs when the molecule releases its energy in the form a fluorescent photon and transitions back to the ground state. Generally, the fluorescence emission characteristics (e.g., spectrum and decay time) are specific to the structure of the molecule rather than depending on the excitation light. Therefore, by analyzing the fluorescence emission, one can obtain information specific to the molecule (often called fluorophore). Typical endogenous fluorophores include amino acids (tyrosine and tryptophan), structural proteins (elastin and collagen), and enzyme cofactors (e.g., NADH and FAD). Fluorescence spectroscopy of endogenous fluorophores also has the potential to monitor tumor microenvironments because of their proportional changes during the alteration of biochemical composition, cellular structure, interactions/bonding with other molecules, and tumor metabolism (Ramanujam, 2000).

Steady-state fluorescence spectroscopy measures fluorescence intensity, which is proportional to fluorophore concentration. In principle, multiple fluorophores emitting at different spectral regions may be resolved qualitatively and even quantitatively. The measured intensity, however, is often subject to several acquisition artifacts such as detection geometry, scatters, chromophores, and photobleaching. Combined with the broadband nature of the autofluorescence (i.e., overlapping spectra) and unknown types of fluorophores contributing to the measured signal, it is difficult to accurately determine the types of fluorophores and their relative contributions. In contrast, fluorescence lifetime (τ) is relatively independent of the aforementioned intensity artifact. More importantly, it is sensitive to intermolecular interactions, changes of adjacent microenvironment, and allows separation of spectrally overlapping molecules as an added source of contrast (Suhling et al., 2005). For instance, NADH exhibits short and long lifetime components depending on its binding status with proteins. The relative ratio of free to bound NADH in tumors can be measured by the changes of the average fluorescence lifetime of NADH (Skala et al., 2007b). Thus, the fluorescence intensity and lifetime of NADH become biomarkers for monitoring a tumor’s environment. Spectral characteristics and properties of endogenous fluorophores have been reviewed comprehensively (Wagnieres et al., 1998; Ramanujam, 2000). The instrumentation of fluorescence-based biopsy technologies has been well summarized recently by Kittle et al. (2016). Fluorescence-based optical biopsy techniques have been studied in oral carcinoma (Skala et al., 2007a; Sun et al., 2013; Cheng et al., 2014), upper GI tract (Kiesslich et al., 2006; Kara et al., 2007; Dunbar et al., 2009; Dunbar and Canto, 2010; Wallace et al., 2010; Bertani et al., 2013; Boerwinkel et al., 2015), colon (Ramanujam, 2000; Buchner et al., 2010; Coda et al., 2014), lungs/bronchi (Zellweger et al., 2001), brain (Gebhart et al., 2007; Kantelhardt et al., 2008, 2016; Butte et al., 2010; Lin et al., 2010; Sun et al., 2011; Valdés et al., 2011; Nie et al., 2016; Du Le et al., 2017), skin (Galletly et al., 2008; Nie et al., 2013), and bladder (Sonn et al., 2009).

Time-resolved fluorescence (TRF) provides additional contrast and is sensitive to the fluorophores’ microenvironment. Due to the technical challenges in measuring fast fluorescence decays in the nanoseconds range, only a limited number of TRF studies have been reported for GI applications [esophagus (Glanzmann et al., 1999; Pfefer et al., 2003), colon (Mycek et al., 1998)]. In the past 10 years, a number of groups have developed clinically-compatible instruments and used them to study neoplastic lesions at different stages (Mycek et al., 1998; Glanzmann et al., 1999; Pfefer et al., 2003; Park et al., 2010; Fatakdawala et al., 2013; Jabbour et al., 2013; Cheng et al., 2014; Coda et al., 2014; Pande et al., 2016). Using a streak camera-based TRF instrument, Glanzmann et al. (1999) demonstrated that when excited at 337 nm, normal and cancerous esophageal tissue fluorescence spectra significantly overlap such that the two cannot be differentiated. Their fluorescence lifetimes show significant differences: normal tissue fluorescence decays faster than cancerous tissue in 375–400 nm and slower between 465 and 485 nm. These findings suggest that time-domain features are an additional source of contrast when dealing with broad band autofluorescence signals. No statistics-based classification was provided in this work as data from only one patient was used for demonstration purposes. Mycek et al. (1998) used a pulse sampling technique to study colon lesions in a 17-patient cohort. The average lifetime of non-cancerous polyps is 10.5 ns, which is statistically different from cancerous polys lifetime of 9.3 ns. Using a similar approach, Pfefer et al. (2003) conducted an *in vivo* study on esophageal tissue with 337 and 400 nm excitation in 37 patients. Linear discriminate analysis (LDA) was used in classification to achieve a sensitivity of 74% and a specificity of 67–85%. The classification was completed based on spectral features and adding bi-exponential lifetime parameters, but it did not lead to better classification outcomes, even when the decay of dysplastic tissue is faster than normal tissue. Table 2 summarizes selected optical biopsy studies using steady-state and TRF.

**TABLE 2:** Steady-state (SS)/time-resolved fluorescence (TRF) approaches for GI optical biopsy.

**Location**	***In vivo*/*ex vivo*/ *in vitro***	**Human/animal**	**Excitation**	**Emission**	**Lifetime**	**References**
Oral (including saliva glands)	*Ex vivo*	Human	455 nm	RGB Filter	N/A	Muldoon et al., 2012
	**Ex vivo**	Human	250–540 nm (SS) 460 nm (TRF)	270–750 nm (SS) 625 nm (TRF)	Tumor: 13.4 ns, 3.2 ns Normal: 9.6 ns, 2.7 ns	Yuvaraj et al., 2014
	*Ex vivo*	Human	280 nm 310 nm	350 nm	Pre-malignant: 4.7 ns Normal: 5.0 ns	Kanniyappan et al., 2016
	***In vivo***	Hamster	337 nm	360–600 nm 390/450 nm (TRF)	5.7 ns at 390 nm 4.8 ns at 450 nm	Sun et al., 2012
	**In vivo**	Hamster	355 nm	390 nm 452 nm >500 nm	Normal/tumor 5.67 ns/5.43 ns 4.67 ns/2.62 ns 4.34 ns/5.62 ns	Jabbour et al., 2013
	*In vivo*	Hamster	355 nm	390 nm 452 nm >500 nm	3.91 ns 2.36 ns 1.92 ns	Cheng et al., 2014
Esophagus	*In vivo*	Human	405 nm 337/400 nm 337 nm	475–675 nm 530–570 nm 375–400 nm 465–485 nm	N/A 3.36/3.74 ns 3.74/3.27 ns	Glanzmann et al., 1999; Pfefer et al., 2003; Boerwinkel et al., 2015
Stomach	*In vivo*	Human	405 nm	>430 nm	N/A	Murayama et al., 2012
Gastric	*Ex vivo*	Human	355 nm	>375 nm	Tumor: 3.73 ns Mucosa: 3.15 ns	McGinty et al., 2010
Metastasis gastric	*Ex vivo*	Human	405 nm	>430 nm	N/A	Koizumi et al., 2013
Colon	*Ex vivo*	Mouse	785 nm	800–850 nm	N/A	Iftimia et al., 2012

Besides single-point measurements, fluorescence imaging can be achieved by raster scanning as in confocal laser endomicroscopy (CLE; Kiesslich et al., 2006; Canto, 2010) or through an imaging fiber bundle as in high-resolution microendoscopy (HRME) (Muldoon et al., 2007, 2010). Current confocal endoscopes permits cytology-comparable resolution using miniaturized confocal optics placed at the distal end of the optical fiber. The depth of imaging acquisition is up to 250 μm (EC3870CILK, Pentax, Tokyo, Japan), which allows for microscopic visualization of mucosal layers and capillaries (Canto, 2010). Kiesslich et al. (2006) have shown that confocal endoscopy is capable of differentiating BE and adenocarcinoma with the use of fluorescein for visualizing cellular and vascular patterns. The Mainz Confocal Barrett’s Classification Criteria were subsequently developed based on the morphological characteristics to stage BE. This classification system achieved high diagnostic accuracy (97.5%) and successfully enabled targeted HGD biopsy, while it is still considered to be highly dependent on intra-observer agreement (Muldoon et al., 2010). In addition to the upper GI tract, CLE has also been demonstrated to have higher sensitivity in detecting colorectal polyps (91%) compared to histopathology, with comparable specificity (Buchner et al., 2010), and to differentiate normal, low, and high-grade bladder neoplasia based on cellular architecture and pleomorphism (Sonn et al., 2009). More recently, fluorescence lifetime imaging microscopy (FLIM) has been under extensive investigation for oral cancer diagnosis (Muldoon et al., 2012; Sun et al., 2012; Jabbour et al., 2013; Cheng et al., 2014; Yuvaraj et al., 2014; Kanniyappan et al., 2016; Malik et al., 2016; Pande et al., 2016). Many of these approaches used a rigid scope with raster scanning capabilities and FLIM is combined with other scanning-based modalities including optical coherent tomography (OCT) (Park et al., 2010) and ultrasound (Sun et al., 2012). In these studies, the classification is mostly based on fluorescence lifetime from a limit number of spectra bands. The addition of other modalities is often used for validating the classification results rather than for providing an additional source of contrast. The 2D imaging capability is a powerful feature allowing defining the margins of the lesions.

### Scattering-Based Techniques

Light scattering characteristics are determined by tissue optical properties, such as concentrations of absorbers (e.g., hemoglobin as an indicator of microvasculature changes at focal dysplasia lesions or intestinalized epithelium) and scatterers (e.g., solid tumor). For example, diffuse reflectance spectroscopy (DRS) provides local tissue optical properties (i.e., absorption, scattering, and anisotropy), which can be used to correct measured fluorescence emission (Du Le et al., 2015). In contrast to elastic scattering properties, Raman spectroscopy measures inelastic scattering events to probe specific chemical components in biological tissue including proteins, lipids, nucleic acids, and water (Le et al., 2009). Therefore, scattering-based techniques represent a sensitive tool to probe changes in structure compositions and have been used alone or combined with fluorescence for detection of dysplasia in oral cavity (De Veld et al., 2005; Schwarz et al., 2008; van Leeuwen-van Zaane et al., 2013; Einstein et al., 2016; Bailey et al., 2017), upper GI tract (Feng et al., 2013; Almond et al., 2014; Bergholt et al., 2014; Douplik et al., 2014; Lariviere et al., 2018), pancreas (Zhang et al., 2017), as well as in colonoscopy (Baltussen et al., 2017). Among these, both Raman spectroscopy (Teh et al., 2010; Feng et al., 2013; Almond et al., 2014; Bergholt et al., 2014), and DRS (Bergholt et al., 2014; Douplik et al., 2014; Lariviere et al., 2018) have been explored for GI cancer diagnosis applications. For *in vivo* applications, Raman spectroscopy is a very specific technique, but it suffers from low signal levels.

Optical coherence tomography (OCT) has been used to obtain cross-sectional images using low coherence light. Similar to the concept of ultrasonic pulse-echo imaging, OCT is based on the scattering events from tissue microstructures due to the mismatch of refractive indices. The structures of the targeted volume can be reconstructed based on the interference patterns between the incident and reflected light (Huang et al., 1991).

Recently, high-resolution OCT has also been developed as a potential tool for imaging stratified layers of esophageal epithelium (Bouma et al., 2000; Pitris et al., 2000; Wojtkowski et al., 2004; Evans et al., 2007; Cobb et al., 2010; Suter et al., 2014), achieving lateral resolution of a few micrometers, axial resolution down to 2 μm in tissue, and a penetration depth of 2–3 mm (Cobb et al., 2010). This allows visualization of tissue architecture such as crypts and blood vessels, and differentiates BE from normal squamous epithelium. The greater imaging depth compared to confocal microscopy is particularly valuable for visualizing submucosal invasion and staging of neoplastic lesions. However, as the contrast from OCT is built upon the mismatch of refractive indices, subcellular organelles may not be visible using this technique, thus further correlation may be required.

### Photoacoustic Imaging

Photoacoustic tomography (PAT) is an emerging technique that has been used in imaging biological tissue. The imaging contrast of PAT is based on the ultrasonic detection of thermal expansion from biological components, such as hemoglobin and melanin (Wang and Hu, 2012). Specifically, a photothermal effect is produced by the energy absorption that occurs after short-pulsed laser illumination of the biological tissue. PAT has been widely used to image from the cellular level to organ specimens with the use of certain contrast agents (Wang and Hu, 2012). An *in vivo* case reported by Kircher et al. (2012) used PAT, MRI, and Raman spectroscopy to investigate the brain tumor margin in a mouse model. In this study, PAT produced a three-dimensional image to localize the position of brain tumor using a nanoparticle contrast agent, thus yielding an improved accuracy in tumor detection. Yang et al. (2015) have reported photoacoustic images of the vasculature of a vertebrate esophagus with the resolution of 190 μm.

### Multimodal Optical Biopsy

As reviewed in previous sections, wide field endoscopic imaging and optical biopsy possess unique and complementary advantages: endoscopic imaging is able to perform large field of view surveillance, while the point optical biopsy helps to demarcate focal and irregular lesions. Combinations of wide field and high-resolution detection have been shown to accurately identify normal tissue and moderate to severe dysplasia (Pierce et al., 2012). Moreover, optical biopsy techniques of different contrast mechanisms provide distinct and complementary information, encompassing tissue compositions (e.g., steady-state fluorescence, diffuse or confocal reflectance, OCT), biochemical microenvironment, and molecular profiles (e.g., TRF, Raman spectroscopy). For instance, fluorescence emission from tissue can be corrected with tissue optical properties when used in combination with DRS, which has demonstrated increased diagnostic sensitivity and specificity in brain tumors (Valdés et al., 2011; Du Le et al., 2017). DRS detected at multiple wavelengths (485, 513, 598, 629 nm) has also been proposed as a complementary tool with upper GI endoscopy to show vascular composition and oxygenation (Douplik et al., 2014). Morphometry analysis has also been used in combination with FLIM to study oral cancer lesions (Park et al., 2010; Jabbour et al., 2013). Studies show OCT allows volumetric mapping of oral epithelial sub-layers and vessels (Park et al., 2010) and reflectance confocal microscopy reveals subcellular resolution (Jabbour et al., 2013). Fluorescence intensity and lifetime detected at multiple spectral bands showed decreased collagen content and increased metabolism (NADH, FAD, and porphyrins) of the lesions. To further increase the depth of visualization, Marcu’s group utilized ultrasonic backscatter microscopy (UBM) with fluorescence lifetime to assess lesions involving invasion deeper than 2 mm with 25–50 micron resolution, which further allowed for accurate staging of the malignancy (Sun et al., 2012). Moving forward, photoacoustic imaging that shares the same transducer with UBM was added into this bimodal system, and has successfully demonstrated changes in vascularization and mucin accumulation in neoplastic epithelia (Fatakdawala et al., 2013). Taken together, multimodality optical imaging therefore represents an emerging and powerful concept to obtain synergistic interrogations of precancerous and neoplastic lesions (Bedard et al., 2010).

### Future Perspectives for Clinical Translation

The optical biopsy applications reviewed above indicate its unique merit in assisting diagnosis in the GI tract. Of note, the complexity of *in vivo* microenvironment, such as existence of intrinsic fluorophores (e.g., autofluorescence), variation in light propagation, and blood perfusion (e.g., oxygenation), will need substantial multidisciplinary work to delineate the measured results *in vivo*. Specifically, efforts should be made in the following areas for clinical translation:

(i) Robust data analysis will be needed to decompose substantial biological variables that could be present in the measured data. For example, fitting accuracy of the fluorescence decay may be reduced when trying to resolve more multiple exponential components. Such a challenge could be overcome with instrumentation development to acquire data with higher signal-to-noise ratio (SNR), aided by the development of more efficient curve fitting algorithms. The spectral-resolved analyses presented in this work will, however, be a good add-on to help resolve and interpret multiple sources of fluorophores.
(ii) Tissue optics will affect light propagation to the target and back to the detector. This can be improved by modeling light transport.
(iii) The ongoing development of microendoscopy will also facilitate translation of optical biopsy. Overall, the generalization to clinical applications will be feasible given the intensive developments in the field of microendoscopy, tissue optics modeling, and computation.

## Fluorescence Lifetime and Spectra-Based Optical Biopsy of Upper GI Tissue

As shown in Table 2, there are limited studies on fluorescence spectroscopy in the esophagus, stomach, and small intestine. Here we report an *ex vivo* clinical study using TRF spectroscopy to investigate the fluorescence characteristics of different tissue types in the upper GI tract, especially for the esophagus, stomach, and duodenum. The aims of this study are: (1) assessing the feasibility of rapid acquisition of TRF spectra from fresh GI mucosal specimens and (2) identifying spectroscopic signatures that differentiate normal mucosa from the esophagus, stomach, and duodenum. Such data will serve as a baseline for future studies on diagnostics value of fluorescence based optical biopsy for GI cancer diagnosis.

The study protocol has been approved by the Hamilton Integrated Research Ethics Board (HiREB), which covers both McMaster University and Hamilton Health Sciences. A total of 28 patients were recruited in this study when they were scheduled for upper GI endoscopy examination. For each patient, specimens from different locations in the upper GI (esophagus, stomach body and antrum, and duodenum) were investigated in the endoscopy theater within 5–30 min following excision. During the measurements, the specimens were kept in a Petri dish and remained hydrated with saline. The specimens were later stored in formaldehyde for further histopathological investigation.

### Experiment Protocol

**FIGURE 1:**
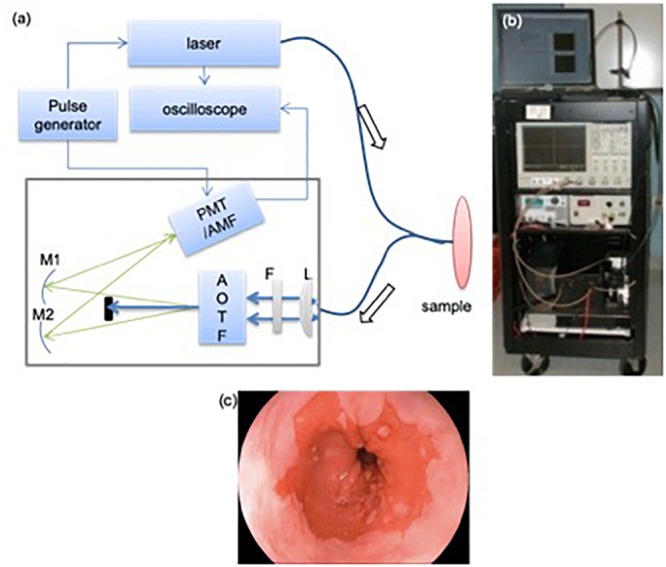
TRF instrument used for the clinical study **(a)**: A schematic and picture of TRF spectroscopy system. A bifurcated fiber optics probe was used to collect signal. AOTF: acousto-optical tunable filter; L: plano-convex lens; F: long-pass filter (cut-off at 360 nm); M1/M2: mirrors; PMT: micro-channel plate photo-multiplier tube; AMF: amplifier. Bottom: endoscopic image showing the extent of the BE region. **(b)** A photograph of the optical biopsy system. **(c)** A representative endoscopic image of the esophagus.

During the endoscopic procedure, specimens from different locations in the upper GI (esophagus, stomach body, antrum, and duodenum) were obtained using standard tissue biopsy forceps, passed through the working channel of the endoscope. The choices of these specimens were completely driven by clinical needs: some are polyps requiring histology analysis; most were randomly taken. The excised specimens were placed on a petri dish hydrated with saline. These specimens were then measured using a clinical compatible TRF spectroscopic instrument. Most of the specimens were measured as they were taken out of the endoscope. Typical time between excision and measurements ranged between 5 and 30 min. The longer intervals were the result of procedural logistics which prevented the fluorescence measurements to be conducted concurrently. Figure 1 shows the schematics of the multimodality biopsy system. Details of the instrument can be found elsewhere (Yuan et al., 2009). Briefly, a picosecond (300 ps/FWHM, 3 μJ/pulse), Nd:YAG laser at 355 nm was used as excitation. A fiber-optic probe was place at 0.5 cm above the specimen at 45° to reduce the back scattered laser light. The excitation fluence on the tissue surface is 1.24 μJ/mm^2^ with an illumination area of 2 mm^2^. This pulse energy meets the safety requirement for the biological tissue and avoids the photobleaching of the sample (Marcu et al., 1999). The autofluorescence emission spectra and decay were measured between 360 and 550 nm at 5 nm intervals. Ten decay curves were averaged at each wavelength to improve SNR. TRF data acquisition time for each location was 4 s.

### Data Processing

The SNR at the peak emission wavelength of fluorescence emission was in the range of 4–20 dB with an average of 10 dB. Intrinsic fluorescence function (IRF) was recovered by numerical deconvolution of the measured laser input from the measured fluorescence signal. Due to the low SNR of the fluorescence decay signal, an oscillation was introduced by the over fitting. Therefore, a Laguerre-based deconvolution method was used to retrieve the IRF rapidly (Jo et al., 2004; Liu et al., 2012). The averaged lifetime (τ_avg_) is calculated based on Eq. 1:

(1) $$\tau_{a\,v\,g}=\frac{T\cdot \sum_{n=0}^{N}n.h\,\left(n\right)}{\sum_{n=0}^{N}h\,\left(n\right)},$$

where *h(n)* is the IRF fitting results and *N* is measurement data points. The integrated intensity was also calculated by averaging the IRF over the observed time scale. These two parameters at each wavelength were considered in the following data analysis.

The features derived from TRF spectroscopy (I_λ_, τ_λ_) at each wavelength are used to distinguish tissue types. In total, 37 intensity features and 37 lifetime features were considered. In order to find significant features that could provide best discrimination performance, one-way ANOVA was used to compare the fluorescence parameters at each wavelength λ for different tissue types as defined by diagnostic results. A *p*-value of < 0.05 was assumed to indicate statistical significance. Moreover, Pearson correlation coefficient was also used to determine the correlation between the features selected by ANOVA to reduce the feature size. If the correlation coefficient is > 0.5, these features could be considered as two different features. Otherwise, only the feature with small *p*-value will be kept.

After decreasing the feature dimension and removing correlated features, we applied a classification method to differentiate the tissue types using the significant features. The forward feature selection method was used to select the best features from each feature catalog. Support vector machine (SVM) (Hsu et al., 2010) was used in this study. In order to estimate the performance of the classification results, the leave-one-out cross-validation method was used to calculate the error between classification results and prediction groups. Finally, the sensitivity and specificity were calculated for each tissue type after applying the classification methods to evaluate the classification performance. All statistical analysis, data processing and classifications were conducted using MATLAB.

## Results

Four tissue types of upper GI tract were examined including esophagus, stomach body, stomach antrum, and duodenum. Both normal and abnormal specimens from esophagus were measured. Each of the biopsy specimens was classified by a pathologist and the results are listed in Table 3. The normal tissue data include duodenum (*N* = 27), stomach antrum (*N* = 5), stomach body (*N* = 19), and esophagus (*N* = 16). In all, 11 specimens were classified as abnormal, including esophagitis (*N* = 8) and dysplastic Barrett’s esophagus (*N* = 3).

**TABLE 3:** Diagnosis results from a pathologist.

**Tissue types**	**Diagnosis results**	**Sample numbers**
Duodenum	Normal	27
Antrum	Normal	5
Stomach	Normal	19
Esophagus	Normal	16
	Esophagitis	8
	Dysplasia BE	3

**FIGURE 2:**
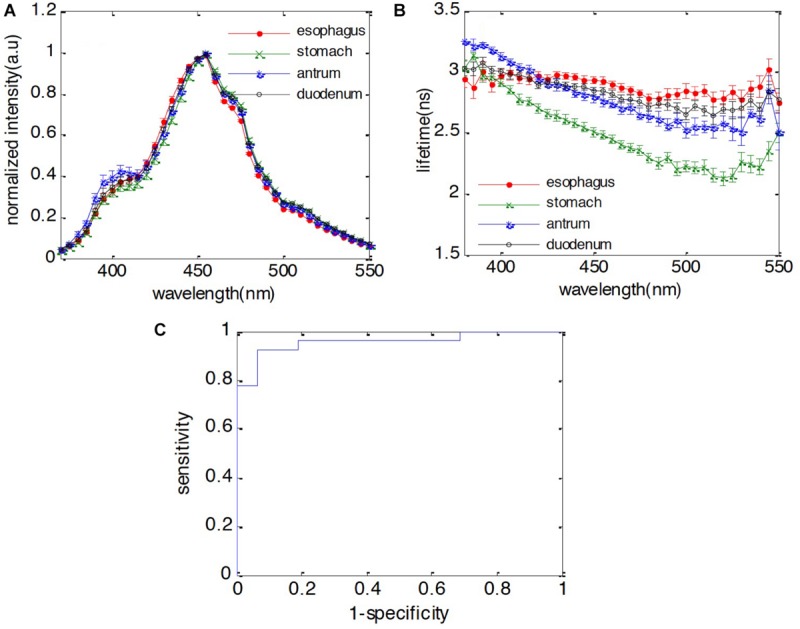
Fluorescence characteristics of duodenum, antrum, stomach body, and esophagus. **(A)** Spectra; **(B)** lifetime; **(C)** receiver operating characteristic (ROC) curve of classification between duodenum and esophagus using both spectral and lifetime features. The area under curve (AUC) is 0.95.

The steady-state fluorescence spectra and lifetime of the four normal tissue types over the measured wavelength region (370–550 nm) are shown in Figure 2. The four tissue types have almost the same emission spectral shape peaking at 455 nm, whereas their lifetime values are different. The lifetime of the stomach body is significant shorter than the rest of the group, which have significant overlap across the measured spectral region. Generally, all four tissue types exhibit longer lifetime (∼3 ns) in the UV and gradually decreases to the green (∼520 nm). Both stomach tissue types (stomach body and antrum) exhibit similar decreasing trend from 360 to 520 nm, while the lifetime of stomach body is notably shorter (3.13 ± 0.03 ns at 380 nm to 2.12 ± 0.06 ns at 520 nm) than that of antrum (3.25 ± 0.02 ns at 385 nm to 2.58 ± 0.06 ns at 520 nm). The lifetime of normal esophagus has the minimum variation across the measured spectrum, while the lifetime of duodenum decreased slightly to 2.79 ± 0.05 ns at 520 nm.

When performing surveillance for esophageal or gastric malignancies, the presence of IM is considered to be a key finding in the metaplasia-dysplasia-carcinoma transformation process. For instance, normal esophageal tissue consists of stratified epithelium cells while IM is characterized by columnar cells similar to those in the small intestine. In this study, one objective is to investigate whether the fluorescence features of duodenum are different from normal esophagus, but similar to BE metaplasia. First, we would like to identify whether the autofluorescence signal can be used to differentiate these two different cell types. By applying the forward selection method with SVM classification method with leave-one-out cross validation, we observed that spectral features at 475, 485, and 490 nm and the lifetime features at 385, 395, and 405 nm can be used to differentiate the duodenum from normal esophagus with 96% sensitivity and 87% specificity. The receiver operating characteristic (ROC) curve was calculated for the developed classification model to separate the duodenum from normal esophagus. The area under the curve (AUC) is 0.95, which indicates that the autofluorescence signal is a very promising technology for GI tissue identification. Additionally, stomach body can be differentiated from esophagus with 94% sensitivity and 100% specificity. The antrum can be differentiated from esophagus with 100% sensitivity and 100% specificity. The classification results are presented in Table 4.

**TABLE 4:** Normal tissue classification results.

**Tissue types**	**Sensitivity**	**Specificity**
Duodenum vs esophagus	Normal	87%
Stomach body vs esophagus	Normal	100%
Antrum vs esophagus	Normal	100%

In addition, the same classification method has also been applied to detect the difference between duodenum and BE, which also contains columnar cells as the result of IM. The fact that their fluorescence features are not statistically different demonstrates that duodenum and BE are quite similar in terms of endogenous fluorophores.

**FIGURE 3:**
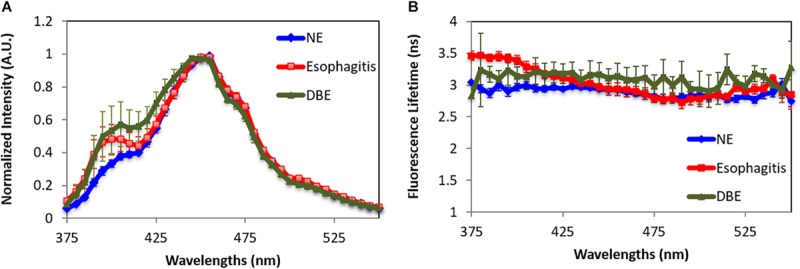
Fluorescence characteristics of normal esophagus (NE), dysplasia Barrett’s esophagus (DBE), and esophagitis tissue. **(A)** Spectra. **(B)** Lifetime. Data are presented as mean ± standard error.

Besides normal tissues in the four locations, a total number of 11 specimens are classified by a pathologist through histology slides to show dysplastic BE or reflux esophagitis. Autofluorescence spectra and lifetime of three different esophagus tissue types are shown in Figure 3. Esophagitis tissue has higher emission in the wavelength range of 375–450 nm compared to the dysplasia BE and the normal esophagus. Moreover, the dysplasia BE group has higher intensity than normal esophagus around 400 nm, which may be caused by different hemoglobin absorption. The lifetime of dysplasia BE group remains the same over the measured spectra with an average lifetime of 3.09 ± 0.11 ns. The lifetime of esophagitis, on the other hand, is longer than both normal esophagus and dysplasia BE below 425 nm with the peak lifetime of 3.46 ± 0.03 ns at 390 nm. Such lifetime differences may be attributed to increased connective tissue (e.g., collagen and elastin) associated with esophagitis.

By using the spectral features in the short wavelength range (375–425 nm) and the lifetime features at 385, 395, 405, and 415 nm, the esophagitis can be differentiated from normal esophagus tissue with 100% sensitivity and 93% specificity. Our data cannot sufficiently differentiate esophagitis from BE. Besides similarities in both spectral and lifetime features, this is attributed to the small number of samples in both groups.

## Discussion and Conclusion

The autofluorescence spectra and lifetime of the different upper GI tissues were investigated in this pilot study on a small set of *ex vivo* specimens. Although these are not *in situ* experiments, the measurements were performed on fresh specimens in the endoscopy theater soon after excision. Hence, the results are believed to be representative enough in retrieving major fluorescence components present *in vivo*.

The fluorescence characteristics including the normalized intensity and average lifetime of duodenum, esophagus, and stomach body are presented. We found four tissue types have similar emission spectra with an emission peak at 455 nm, whereas their lifetime values are different. We are able to differentiate the tissue types based on combined fluorescence spectral and lifetime features. The autofluorescence of BE with dysplasia has also been presented. Again, combining fluorescence spectral and lifetime features can differentiate diseased tissue (esophagitis and BE) from normal esophagus. However, due to the small sample size, our results cannot sufficiently classify BE from esophagitis. Of note, autofluorescence imaging has been explored quite extensively mainly based on the loss of collagen in early neoplastic transformation and enhanced cell metabolism that results in reduction of blue fluorescence and an overall red-tinted lesion. The technique alone has been shown to yield a higher false positive rate for detecting high-grade dysplasia and can be easily confused with similar autofluorescence features expressed in inflamed tissue (Curvers et al., 2008). On the contrary, at the even earlier phase (e.g., metaplasia), minor change in autofluorescence may not suffice. In either scenario, multi-modality will always be preferred. The results presented in this work, however, established baseline fluorescence features of upper GI tissue. Using autofluorescence to diagnose malignancy may eventually be performed together as an additional feature to improve the diagnostic accuracy and will be incorporated into the next phase of study. It should be noted that the vascular supply *in vivo* could possibly affect the fluorescence lifetime of the tissue due to quenching effects from oxygen. There are many factors that would change oxygen levels in tissue including blood perfusion in tumor, occlusion of vessels, reduced vasculature within the tumor mass, or the increased angiogenesis in the tumor tissue. These changes in fluorescence lifetime will be further correlated with steady-state images (e.g., autofluorescence imaging, NBI, high resolution confocal endoscopy), histopathology validation, and the lifetime measurements will be decomposed to evaluate the potential contributions of intrinsic fluorophores and quenchers.

The current work used a TRF instrument to perform measurements on *ex vivo* specimens with near real-time data acquisition. Such system design can be adopted for *in vivo* measurements through two routes: (1) using a fiber bundle through the working channel of an endoscope/colonoscope and (2) integration into CLE. The formal approach is easy to incorporate but would only serve as a proof-of-principle test due to the time required to switch between the fiber probe and the biopsy tool. Our next phase of the project will focus on the development of a multi-modal endomicroscopy platform to incorporate WLE, AE, CLE, and TRF. Such a platform will allow point-of-care diagnosis followed by tissue ablation that could be performed either using high power laser light or photosensitization (Yeh et al., 2015; Yeh, 2015).

## Data Availability Statement

The datasets generated for this study are available on request to the corresponding author.

## Ethics Statement

The studies involving human participants were reviewed and approved by the Hamilton Integrated Research Ethics Board. The patients/participants provided their written informed consent to participate in this study.

## Author Contributions

All authors listed have made a substantial, direct and intellectual contribution to the work, and approved it for publication.

## Conflict of Interest

The authors declare that the research was conducted in the absence of any commercial or financial relationships that could be construed as a potential conflict of interest.
